# Supplementary figures and images for: Mutants of human ACE2 differentially promote SARS-CoV and SARS-CoV-2 spike mediated infection
Source: PLoS Pathog. 2021 Jul 16;17(7):e1009715. doi: 10.1371/journal.ppat.1009715 (PMC8284657; doi:10.1371/journal.ppat.1009715)

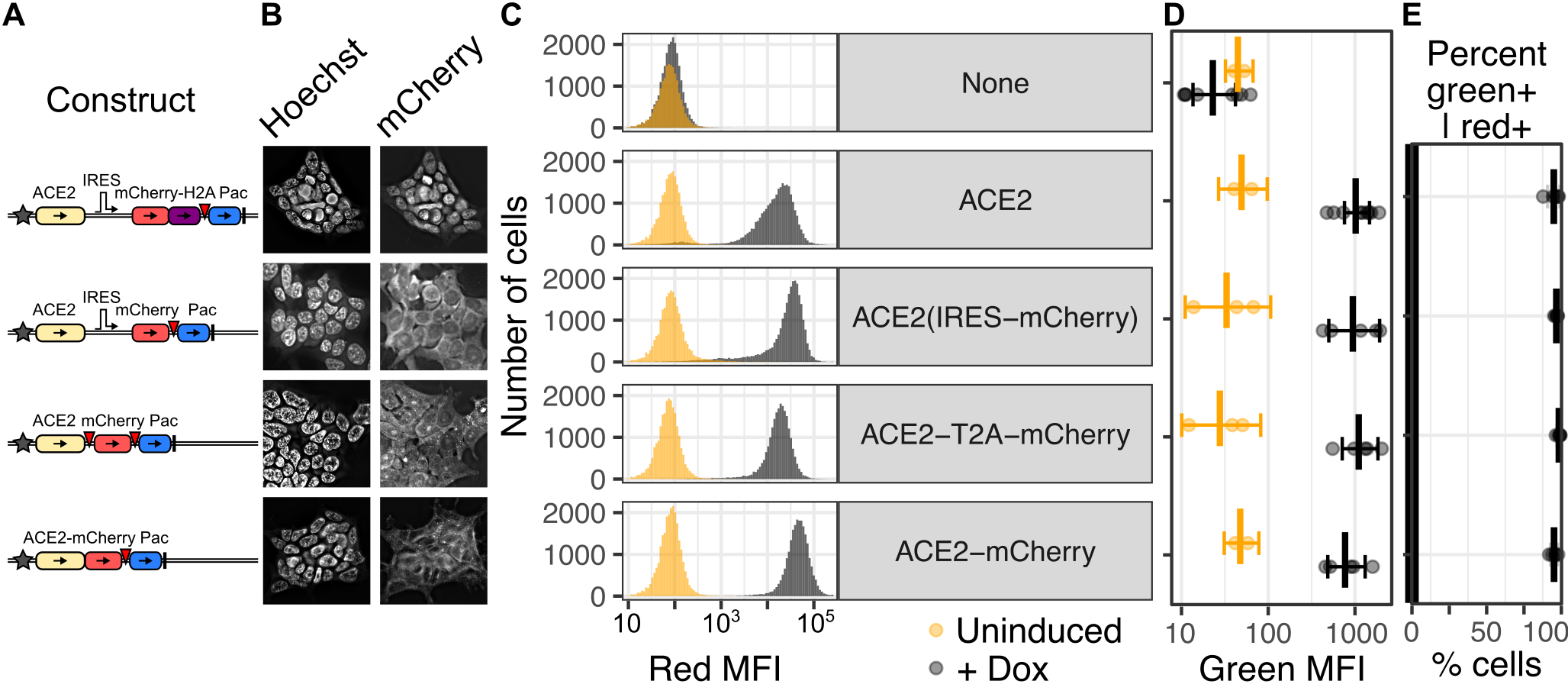

Supplement: S1 Fig — A) Schematics describing each ACE2 expression construct. IRES, Internal Ribosome Entry Site; H2A, Histone 2A; Pac, Puromycin N-acetyltransferase. B) Wide-field fluorescent imaging of ACE2 overexpressing cells, with nuclei stained with Hoechst 33342 (left) and intracellular mCherry distribution (right). C) Representative single-cell distributions of red mean fluorescence intensities, as captured by flow cytometry, in cells left uninduced (orange) or induced to express ACE2 from the Tet-inducible promoter using 2 μM doxycycline (black). D) Replicate staining of cell-surface ACE2 using SARS-CoV-2 RBD-sfGFP. Vertical bars denote the means calculated from the geometric means from each individual replicate. N = 2 and 8 for uninduced and induced cells, respectively. Error bars denote 95% confidence intervals. E) Percentages of mCherry+ cells that also stain for cell-surface ACE2, using SARS-CoV-2 RBD-sfGFP. N = 5, error bars denote 95% confidence intervals. (TIF) [file ppat.1009715.s001.tif]

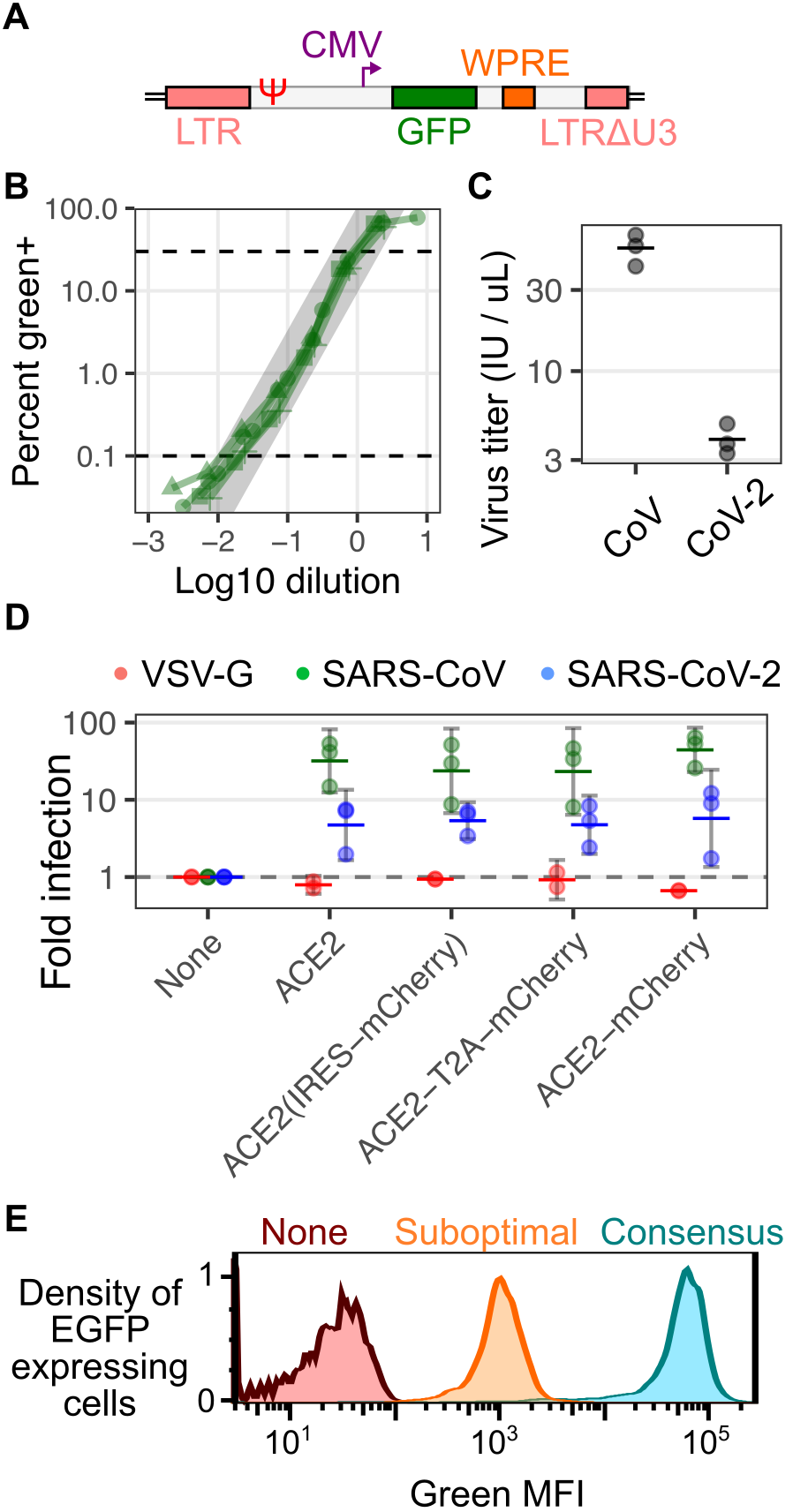

Supplement: S2 Fig — A) Schematic showing a self-inactivated, third-generation lentiviral vector encoding GFP behind a CMV promoter. B) Percentage of green+ cells detected with various dilutions of SARS-CoV spike -pseudotyped green lentiviral vector inoculum. Data from three independent replicates are shown. The gray bar denotes a slope of 1.5. C) Pairwise comparisons of titers derived from equal inocula of SARS-CoV and SARS-CoV-2 spike-pseudotyped lentiviruses, from three independent replicates. N = 3. D) Fold infection in cells expressing each of the indicated ACE2 expression constructs, normalized to infection of parental HEK 293T cells. N = 3, error bars denote 95% confidence intervals. E) Representative smooth histograms of EGFP mean fluorescence intensity observed with flow cytometry. Blue corresponds to EGFP translated behind a consensus “GCCACC” Kozak sequence, orange corresponds to EGFP translated behind the suboptimal “CATTGT” Kozak sequence, and red corresponds to background green fluorescence of the parental cell line in the absence of EGFP. (TIF) [file ppat.1009715.s002.tif]

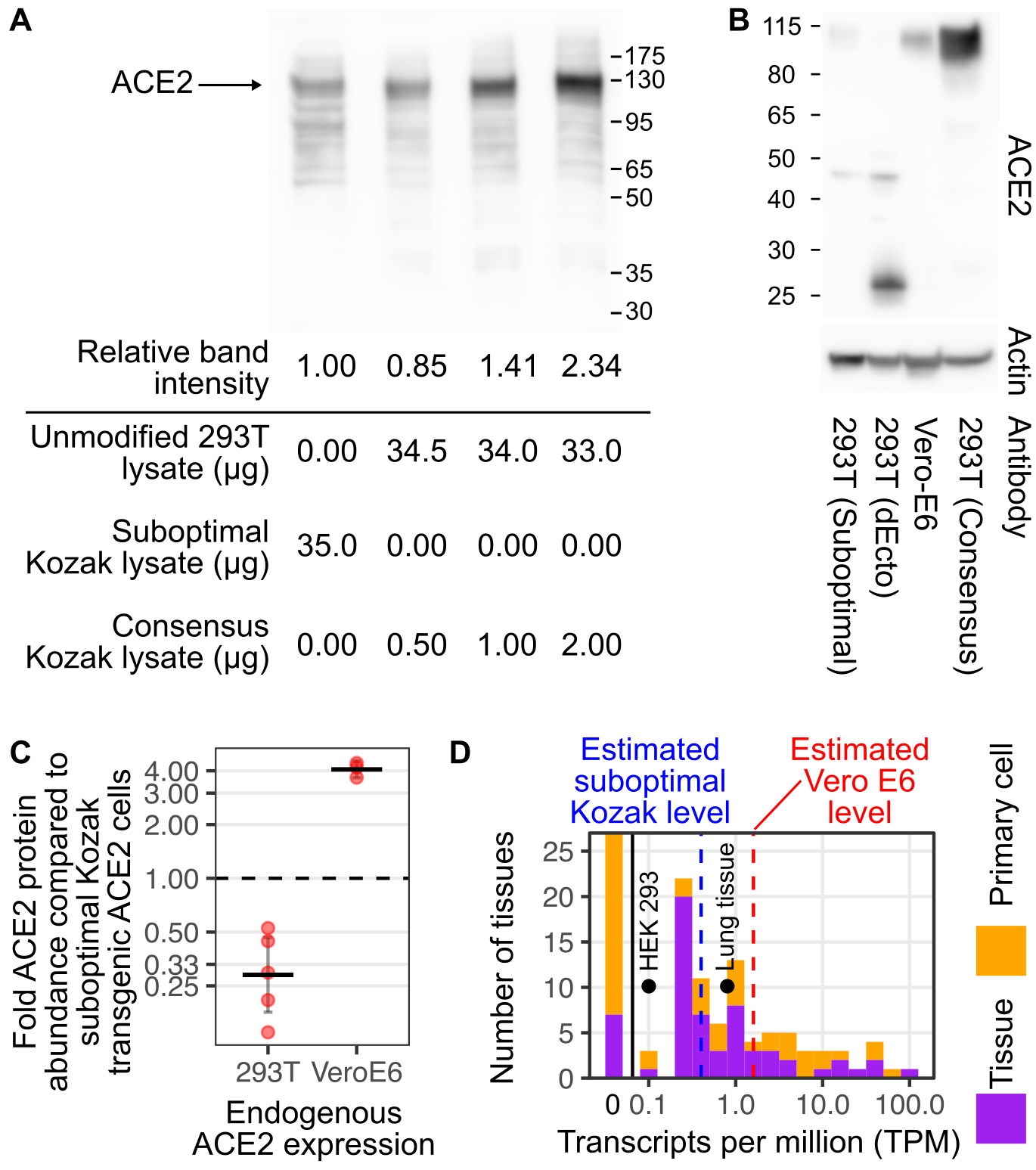

Supplement: S3 Fig — A) To improve quantitation of the relative fold-difference in ACE2 protein expression between the consensus and suboptimal Kozak cells, we electrophoresed small quantities of cell lysates from consensus Kozak ACE2 cells, adding unmodified 293T lysate to normalize for overall protein content, and compared them to undiluted cell lysates from suboptimal Kozak ACE2 cells. B) Western blotting of full-length ACE2 in modified 293T and Vero E6 cells. C) Quantitation of endogenous ACE2 protein abundance in 293T and Vero E6 cells as compared to the suboptimal Kozak ACE2 transgenic cells. The black horizontal line denotes geometric means of replicate blots, and error bars denote 95% confidence intervals. D) Histogram of ACE2 transcripts per million observed in various tissues (purple) or primary cell types (orange) catalogued in GTEx. HEK 293T cells had a 0.1 ACE2 TPM. TPM values corresponding to the relative increase in protein abundance observed in suboptimal Kozak ACE2 transgenic cells (0.4) and Vero E6 cells (1.6) are shown as the dotted blue and red lines, respectively. (TIF) [file ppat.1009715.s003.tif]

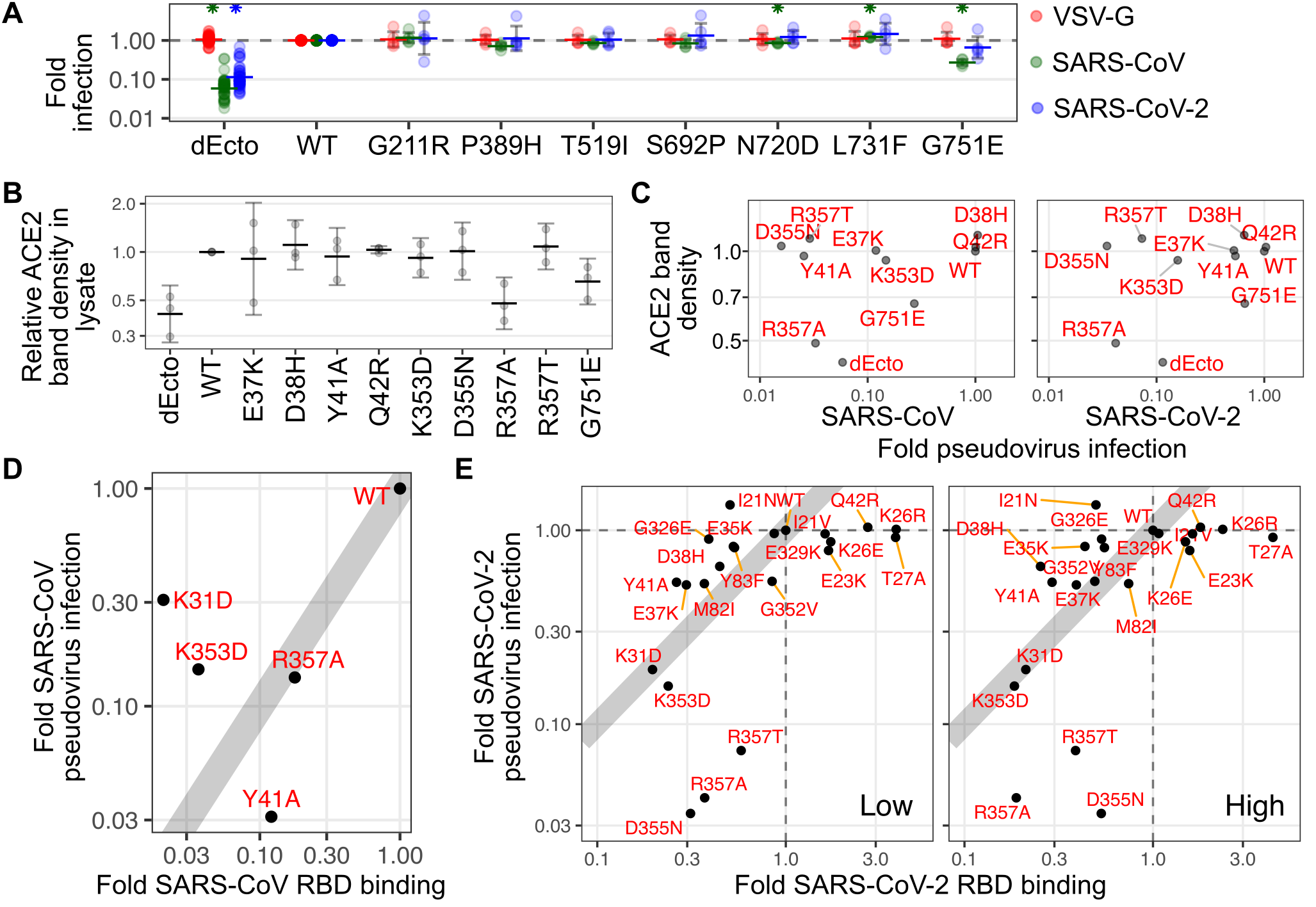

Supplement: S4 Fig — A) Infection of a panel of ACE2 variants of residues outside of the spike interaction interface, which were observed in publicly available human genomics datasets. B) Western blot quantitation of recombinant cells expressing WT ACE2 or its variants behind the suboptimal Kozak. Short horizontal lines are the geometric mean of three independent replicates, and the error bars denote 95% confidence intervals from three replicates. C) Scatterplots comparing WT and Actin-normalized ACE2 Western blotting band intensity to SARS-CoV and SARS-CoV-2 pseudovirus infection results. D) Relative pseudovirus infection rates (y-axis) and published binding to SARS-CoV RBD as assessed by immunoprecipitation (x-axis), for WT ACE2 and its variants. E) Relative pseudovirus infection rates (y-axis) and the value of the published SARS-CoV-2 RBD-sfGFP staining (x-axis) for WT ACE2 and its variants, comparing either the values from the inverse of the low SARS-CoV-2 RBD-sfGFP binding population (nCoV-S-Low; left) or the high binding population (nCoV-S-High; right) sorts. (TIF) [file ppat.1009715.s004.tif]

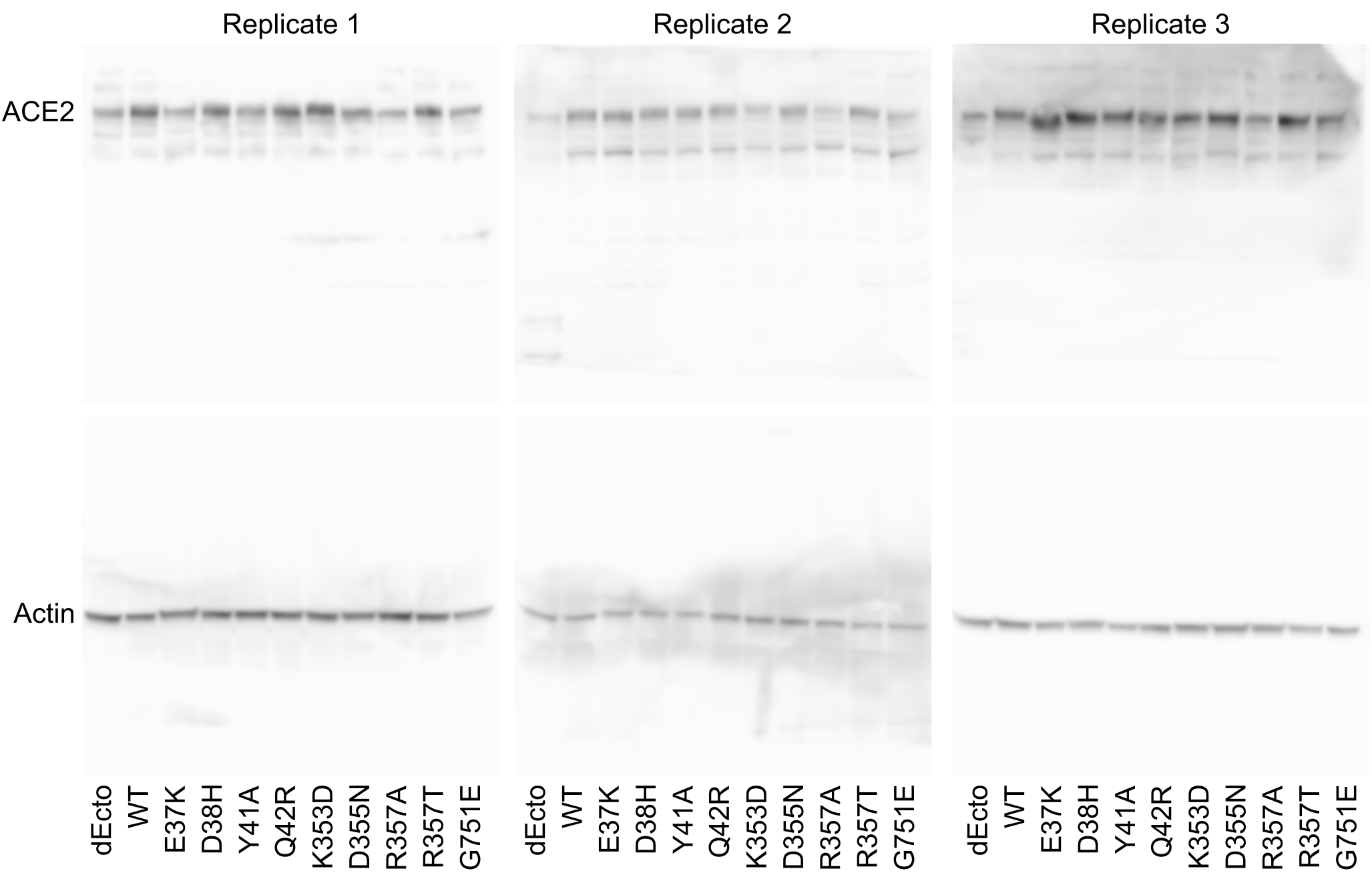

Supplement: S5 Fig — Raw, unscaled western blotting exposures for three replicate experiments assessing the steady-state abundances of the key ACE2 variants tested in the study, blotting for ACE2 protein shown in the top row and blotting for beta-actin as a loading control shown on the bottom row. (TIF) [file ppat.1009715.s005.tif]

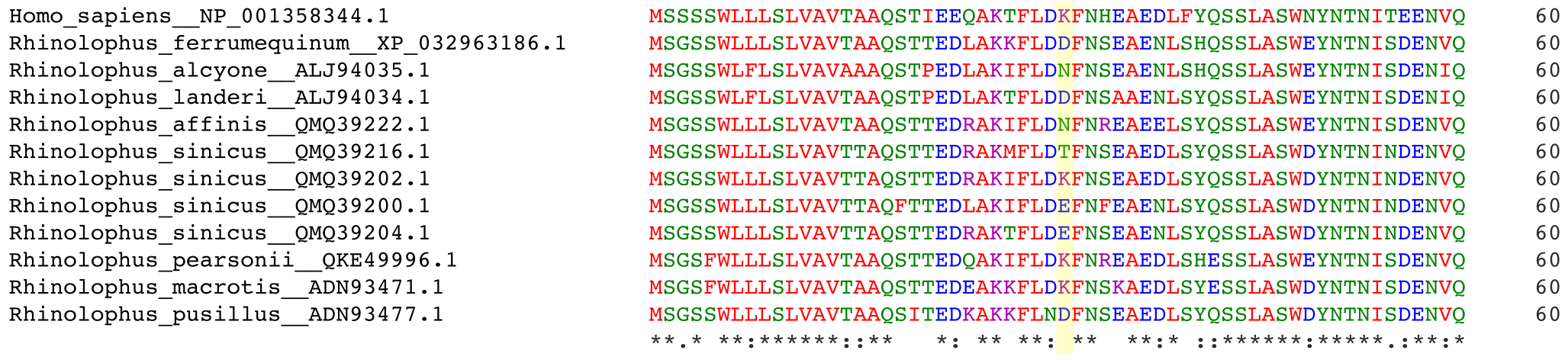

Supplement: S6 Fig — The human ACE2 sequence was used as the query in a Blast search, and ACE2 sequences from the Rhinolophus bat species were collected, with representative members shown in an alignment. The position corresponding to K31 in humans is shown in the partially transparent yellow box. The human ACE2 sequence is shown for comparison. (TIF) [file ppat.1009715.s006.tif]
