# Supplementary material for: Mutants of human ACE2 differentially promote SARS-CoV and SARS-CoV-2 spike mediated infection
Source: PLoS Pathog. 2021 Jul 16;17(7):e1009715. doi: 10.1371/journal.ppat.1009715 (PMC8284657; doi:10.1371/journal.ppat.1009715)
Supplement: S3 Table — (DOCX) [file ppat.1009715.s009.docx]

**S3 Table. List of primers used to create variant constructs used in this study.**

| **Primer names** | **Primer purpose** | **Forward sequence** | **Reverse sequence** |
| --- | --- | --- | --- |
| KAM3316  KAM890 | Create suboptimal Kozak in front of ACE2 | ttataccgcaactacacCATTGTatgtccagctcctcctggctc | gtgtagttgcggtataatctgcgga |
| KAM3320  KAM3321 | Create dEcto variant | cagtccaccattgaggaaagccgtatcaatgatgctttccg | ttcctcaatggtggactgagcag |
| KAM3074  KAM3075 | Create I21N variant | GTCCACCAaTGAGGAACAGGCCAAGACAT | GTTCCTCAtTGGTGGACTGAGCAGCA |
| KAM3076  KAM3077 | Create I21V variant | AGTCCACCgTTGAGGAACAGGCCAAGAC | TTCCTCAAcGGTGGACTGAGCAGCAG |
| KAM3049  KAM3050 | Create E23K variant | ccattgagAaacaggccaagacatttttgga | ggcctgttTctcaatggtggaaccggtc |
| KAM3051  KAM3052 | Create K26E variant | aacaggccGagacatttttggacaagtttaacca | aaatgtctCggcctgttcctcaatggtg |
| KAM3053  KAM3054 | Create K26R variant | acaggccaGgacatttttggacaagtttaaccacg | aaaatgtcCtggcctgttcctcaatggt |
| KAM3055  KAM3056 | Create T27A variant | aggccaagGcatttttggacaagtttaaccacg | caaaaatgCcttggcctgttcctcaatgg |
| KAM3106  KAM3107 | Create K31D variant | ttggacGaCtttaaccacgaagccgaagac | gttaaaGtCgtccaaaaatgtcttggcctg |
| KAM3057  KAM3058 | Create E35K variant | ttaaccacAaagccgaagacctgttctatca | ttcggcttTgtggttaaacttgtccaaaaatgtct |
| KAM3059  KAM3060 | Create E37K variant | acgaagccAaagacctgttctatcaaagttcactt | caggtcttTggcttcgtggttaaacttgtc |
| KAM3092  KAM3093 | Create D38H variant | aagccgaaCacctgttctatcaaagttcacttg | gaacaggtGttcggcttcgtggttaaactt |
| KAM3108  KAM3109 | Create Y41A variant | cctgttcGCtcaaagttcacttgcttcttgga | actttgaGCgaacaggtcttcggcttcg |
| KAM3096  KAM3097 | Create Q42R variant | gttctatcGaagttcacttgcttcttggaa | gtgaacttCgatagaacaggtcttcggcttc |
| KAM306  KAM3062 | Create M82I variant | gcccaaatCtatccactacaagaaattcagaatctca | agtggataGatttgggcaagtgtggact |
| KAM3098  KAM3099 | Create Y83F variant | ccaaatgtTtccactacaagaaattcagaatctca | gtagtggaAacatttgggcaagtgtggac |
| KAM3644  KAM3645 | Create G211R variant | ggagactatgaagtaaatAgggtagatggctatgactacag | atttacttcatagtctcctctccaataatcccc |
| KAM3646  KAM3647 | Create G326E variant | ttcctaatatgactcaagAattctgggaaaattccatgctaac | cttgagtcatattaggaagaccaacagatacaa |
| KAM3065  KAM3066 | Create E329K variant | gattctggAaaaattccatgctaacggaccc | gaattttTccagaatccttgagtcatattaggaa |
| KAM3067  KAM3068 | Create G352V variant | ggacctggTgaagggcgacttcaggatc | cgcccttcAccaggtcccaagctgtg |
| KAM3114  KAM3115 | Create K353D variant | ctggggGaCggcgacttcaggatccttatg | gtcgccGtCccccaggtcccaagct |
| KAM3069  KAM3070 | Create D355N variant | ggaagggcAacttcaggatccttatgtgcac | cctgaagtTgcccttccccaggtcc |
| KAM3116  KAM3117 | Create R357A variant | cgacttcGCgatccttatgtgcacaaaggtga | aaggatcGCgaagtcgcccttcccca |
| KAM3104  KAM3105 | Create R357T variant | cgacttcaCgatccttatgtgcacaaaggtga | taaggatcGtgaagtcgcccttcccc |
| KAM3648  KAM3649 | Create P389H variant | tggcatatgctgcacaacAttttctgctaagaaatggagctaat | gttgtgcagcatatgccatatcatactg |
| KAM3650  KAM3651 | Create T519I variant | ttcgatattacacaaggaTcctttaccaattccagtttcaag | tccttgtgtaatatcgaatgaatgagtaatcatt |
| KAM3652  KAM3653 | Create S692P variant | actgcacctaaaaatgtgCctgatatcattcctagaactgaagttg | cacatttttaggtgcagtgacaaagaaatt |
| KAM3654  KAM3655 | Create N720D variant | gctttccgtctgaatgacGacagcctagagtttctggg | gtcattcagacggaaagcatcattgat |
| KAM3656  KAM3657 | Create L731F variant | ctggggatacagccaacaTttggacctcctaaccagcc | tgttggctgtatccccagaaact |
| KAM3658  KAM3659 | Create G751E variant | tttttggagttgtgatggAagtgatagtggttggcattgt | ccatcacaactccaaaaacaatcagc |
| KAM3338  KAM3339 | Create spike D614G variant | CAGTGCTGTACCAGGgCGTGAACTGTACCGAAGTGCC | CCTGGTACAGCACTGCCACC |
| KAM3677  KAM3678 | Create spike N501Y variant | CGGCTTTCAGCCCACAtATGGCGTGGGCTATCAGC | TGTGGGCTGAAAGCCGTAGG |
