# Supplementary material for: Mutants of human ACE2 differentially promote SARS-CoV and SARS-CoV-2 spike mediated infection
Source: PLoS Pathog. 2021 Jul 16;17(7):e1009715. doi: 10.1371/journal.ppat.1009715 (PMC8284657; doi:10.1371/journal.ppat.1009715)
Supplement: Attachment — Submitted filename: Response_to_reviewers.pdf [file ppat.1009715.s010.pdf]

Note: Reviewer comments are prefixed with # and are colored dark red. Author responses are prefixed with > and colored blue.

Part I - Summary
Please use this section to discuss strengths/weaknesses of study, novelty/significance, general execution and scholarship.

Reviewer #1: Shukla et al. combine cell line engineering and studies of cellular infection to understand how SARS-CoV-2 spike-mediated cellular infection is quantitatively related to ACE2 expression and affinity for the spike RBD. Furthermore, they survey how rare ACE2 variants found in humans impact susceptibility to cellular infection. They show quite strikingly that ACE2 overexpression masks detrimental effects of ACE2 variants on cellular infection. They construct a cell line with reduced ACE2 expression that sensitizes measurements of ACE2 variation, identifying rare variants that hinder SARS-CoV and SARS-CoV-2 entry, and some variants that differentially effect infection by SARS-CoV, SARS-CoV-2, and SARS-CoV-2+N501Y. In several figures, they show quite nice “threshold-like” relationships between ACE2:RBD binding affinity or ACE2 expression with susceptibility to cellular infection. These results are important in large part because ACE2-overexpresing cell lines are used so widely for various questions about SARS-CoV-2 biology. The results also report technological advances for cell line engineering that enable future work as nicely described in the Discussion.

Reviewer #2: The manuscript by Shukla et al. described a procedure in which ACE2 gene was inserted into a specific location in the genomic DNA of 293T cells so that the cells constitutively express ACE2 as a receptor for SARS-CoV and SARS-CoV-2. It further analyzed how the ACE2 variants from human populations support the cell entry of SARS-CoV and SARS-CoV-2. Overall, the concepts of engineering a site-specific integration of ACE2 gene into the cellular genome and analyzing the coronavirus receptor activities of naturally existent ACE2 variants are interesting. I have the following comments.

Part II – Major Issues: Key Experiments Required for Acceptance
Please use this section to detail the key new experiments or modifications of existing experiments that should be absolutely required to validate study conclusions.

Generally, there should be no more than 3 such required experiments or major modifications for a "Major Revision" recommendation. If more than 3 experiments are necessary to validate the study conclusions, then you are encouraged to recommend "Reject".

Reviewer #1: n/a
Reviewer #2: (No Response)

Part III – Minor Issues: Editorial and Data Presentation Modifications
Please use this section for editorial suggestions as well as relatively minor modifications of existing data that would enhance clarity.

Reviewer 1 [Major] Comment 1: The main suggestion that I have that is a genuine critique, is that I would be left more satisfied if I had a bit more contextualization of what “relevant” ACE2 expression levels might be. The authors several times simply refer to a general lack of knowledge on this question – which is perhaps true and is not something I have looked into myself. But my impression (again, I fully admit this is not something I have looked for or found in actual papers), is that tissue-level ACE2 expression is thought to be ‘relatively low’. I understand that’s not something you can hang a hat on, but I guess I just want to emphasize, if there are any

straws of physiological relevance that can be grasped, it would help a lot in understanding whether I should truly ‘care’ about the ACE2-low results more than the highly overexpressed cell line where impacts on cell entry are masked.

> We find this to be a very valuable critique, and have addressed it as follows:

1) We have taken the suggestion proposed by the reviewer in comment three (below), and performed Western blotting comparing the endogenous steady-state ACE2 abundance in unmodified HEK 293T and Vero E6 cells, with the transgenic ACE2 abundance produced with the suboptimal and consensus Kozak constructs. We found the Vero cells possessed a magnitude more total ACE2 protein than unmodified HEK 293T cells, and the suboptimal Kozak cells to have an intermediate level between the two. In contrast, the consensus Kozak ACE2 cells had ACE2 abundances vastly in excess of Vero E6 cells. Thus, the suboptimal Kozak ACE2 cells exhibit an amount of ACE2 abundance comparable to or even slightly less than natively expressed ACE2 from known permissive cells, and likely reflect the lower ACE2 levels observed during natural infection.

2) Furthermore, we used this information to project which primary cell types or tissues may possess enough ACE2 to permit ACE2-dependent entry and infection. We pulled information from GTEx, showing the transcripts per million (TPM) for HEK 293T cells, as well as various primary cells and tissues. Knowing that our suboptimal Kozak cells and the Vero E6 cells had roughly 4-fold and 16-fold increased ACE2 protein abundance than unmodified HEK 293T cells, which had an ACE2 transcript level of 0.1 TPM, we identified cells with TPM values of 0.4 and 1.6 as values that may approximate sufficient endogenous ACE2 expression to permit ACE2-dependent entry. This accounted for 50% and 25% of all tissues and primary cells assessed in GTEx, respectively.

We have accordingly modified the manuscript in the following locations:

Supplementary figure S3, new panels B, C, and D.

Results [Lines 198 to 215]: “To better contextualize the ACE2-dependent infection we observed with the suboptimal Kozak ACE2 construct, we compared ACE2 abundance levels between unmodified HEK 293T cells, which are generally considered non-permissive to SARS-CoV-2 entry, with Vero E6 cells, which are widely considered permissive to SARS-CoV-2 infection, and are commonly used to propagate the virus in BSL3 laboratories [28]. We found that these suboptimal Kozak ACE2 cells exhibited roughly 4-fold more ACE2 protein than unmodified HEK 293T cells (Fig S3B, C). In contrast, the suboptimal Kozak ACE2 cells exhibited roughly 4-fold less total ACE2 protein than Vero E6 cells. With these approximate values in mind, we queried the Genotype-Tissue Expression (GTEx) project portal to estimate which cell types and tissues have ACE2 transcript levels that may render them similar to the level of ACE2-dependent SARS-CoV-2 spike-mediated entry as assessed with our cell models. HEK 293T cells have a small but non-zero value of 0.1 ACE2 transcripts per million (TPM) within the GTEx database (Fig S3D). Projecting a 4-fold increase in ACE2 expression, equivalent to the 4-fold relative increase in ACE2 abundance in the suboptimal Kozak cells, revealed 53% of assessed tissues and cell types had equal or greater ACE2 transcripts. Projecting a 16-fold increase, approximating a relative increase to ACE2 protein abundance seen in Vero E6 cells, revealed 25% of assessed tissues and cell types with equal or greater transcripts. Lung tissue exhibits 0.8 ACE2 TPM, partway between the estimated corresponding levels for suboptimal Kozak and Vero E6 cells (Fig S3D). While these are rough estimates and should be approached with caution, they suggest that a sizable fraction of human tissues and cell types may express enough ACE2 to permit at least low-level ACE2-dependent entry seen with our suboptimal Kozak cells.”

Discussion [Lines 406 to 414]: “Using endogenous ACE2 expression from Vero E6 cells as an additional reference, we found that the suboptimal Kozak ACE2 cells exhibited ~ 4-fold less ACE2 abundance, while ACE2 protein in consensus Kozak cells harbored vastly more. Extrapolating these relative values to tissues and primary cell types assessed in GTEx showed that a sizable fraction of samples had projected ACE2 expression values comparable to our suboptimal Kozak ACE2 HEK 293T cells. Importantly, this rough estimate is only for ACE2-dependency during entry, and other factors such as availability of Cathepsin or TMPRSS2 proteases are unaccounted for. Furthermore, this estimate is only relevant to entry, and does factor in replication within cells, or immune cells. Regardless, this estimate may better contextualize the range of cells and tissues that may become infected, and help steer future studies.”

Reviewer 1 [Major] Comment 2: One place I can think to point toward for discussing “physiological relevance” is studies on antibody neutralization. For example, in a recent preprint (https://doi.org/10.1101/2021.04.03.438258), the antibody S309 is shown to have incomplete and reduced neutralization in ACE2-high cell lines compared to cell lines expressing lower levels of ACE2. S309 demonstrates efficacy in animal models (Fc-independent) as well as protective efficacy in its clinical trial as VIR-7831 (though this in theory could be neutralization-independent functions, though neutralization is still probably at play). This should be confirmed in the literature, but I believe antibodies to the spike NTD similarly do not neutralize (?) in ACE2-high cell lines despite neutralizing in ACE2-low cell lines and protecting in animal models. These observations would therefore suggest that the ACE2-low cell lines were more “representative” of in vivo and could potentially be discussed.

> This is a very useful observation, and we have mentioned this phenomenon in the discussion.

Discussion [Lines 422 to 426]: “Recent studies have revealed differing efficiencies of antibody neutralization depending on the amount of ACE2 on target cells. For example, antibodies S309 and S2X333 exhibited impaired neutralization of SARS-CoV-2 spike pseudoviruses in ACE2 overexpressing 293T cells as compared to other cell lines such as Vero E6 cells[47]. Thus, understanding and precise control of ACE2 abundance in model systems may be critical for accurate measurement of biological phenomena surrounding spike-mediated entry.”

Reviewer 1 [Major] Comment 3: Alternatively (and I only suggest this because I see Vero E6 cells are already at the authors’ disposal per the methods), would it be helpful to detect ACE2 levels in Vero E6 cells by Western in comparison to the ACE2-engineered cell lines? Since this would be a ‘native cell’ expression level to which to compare engineered cells (though I do not know the history of Vero E6 cells and if its ACE2 expression level would be expected to be at least somewhat representative of the original tissue).

> We have taken the reviewer’s suggestion, and have Western blotted Vero E6 cells, which helped us perform additional analyses as described above. Please see the modified supplementary Figure 3 and new text described in the response to comment 1, above.

Reviewer 1 Minor Comment 1: I had some difficulty parsing the one Figure Legend I tried to parse. Fig. 1D-F: I think figure legend text or annotation on plot is missing for explaining the difference between induced and uninduced cells as black versus yellow coloring? 1G legend incomplete: geometric mean of what? (Once again, can deduce but the axis titles maybe aren’t super obvious to all readers.) 1H legend, “Fold infection of ACE2 expression” typo? I didn’t read the remaining legends as carefully (as it is clear what is happening in each figure alongside the text – which is great), but probably all figure legends should be checked for similar levels of self-sufficiency and clarity)

> Thank you for noticing these inaccuracies in Fig 1 legends D through H. We have fixed these legends, which now read:

“D) Representative mCherry fluorescence distribution of ACE2-recombined cells, as captured by flow cytometry, in cells left uninduced (orange) or induced to express ACE2 from the Tet-inducible promoter using 2 μM doxycycline (black). E) Geometric mean of green fluorescence of SARS-CoV-2 RBD-sfGFP -stained ACE2 expressing cells. n = 2 and 8 for uninduced and induced cells, respectively. Error bars denote 95% confidence intervals. F) Representative scatterplot of correspondence of mCherry fluorescence and RBD-sfGFP staining of ACE2 recombinant cells. The green line denotes the linear correlation between red and green MFI for the double-positive population. Pearson’s r^2 = 0.44. G) Percent of mCherry positive cells that were also positive for SARS-CoV-2 RBD-sfGFP staining, for 5 repeat staining experiments. H) Fold pseudovirus infection of ACE2 overexpressing cells, normalized to infection of parental HEK 293T cells. n = 3 for SARS-CoV and SARS-CoV-2 spikes, error bars denote 95% confidence intervals.”

Furthermore, we checked the remaining figure, and have made a number of small changes highlighted in the marked-up revised document.

Reviewer 1 Minor Comment 2: Starting line 227: these ‘variants’ that are being discussed – by comparison to the paragraph starting on line 236, my impression is that these are not human variants, but rather specifically chosen mutations – perhaps from structural reasons? In my reading of the paper, I was led to think at first this meant these were human variants. Perhaps if they are not, they should just be referred to as ‘mutants’.

> We are somewhat used to the convention from human functional genomics, where every conceivable protein-coding change in a human protein is generally referred to as a “variant”, so that individuals who possess such a germline variant are not indirectly referred to as “mutants”. That said, we realize this is a manuscript meant for the virology community rather than the functional genomics or clinical genetics communities, and thus acknowledge that the word “mutant” is perhaps more conventional. But as we now more clearly note in the manuscript, the vast majority of the variants (24 of the 28) that we test are possible through single nucleotide variation, and thus have opted to keep the term “variant” out of respect for the individuals who may harbor them now or may be born with them in the future.

Reviewer 1 Minor Comment 3: Is there any estimate of how the spike density on PV relates to density on SARS2 virions? Perhaps these results suggests that expression from the spike side of the coin is also important to think about in future study?

> We are yet to see a clear comparison of SARS-CoV-2 spike density on pseudoviruses as compared to SARS-CoV-2 virions. At least with pseudovirions, SARS-CoV-2 spike variant D614G is thought to increase spike density as compared to the WT (Wuhan) virus, as shown by Michael Farzan and Hyeryun Choe’s groups (Zhang et al, 2020, Nature Communications; PMID: 33243994), although we are not aware of additional studies that further elaborate on this. We did not see a difference in ACE2 variant between the WT (Wuhan) and D614G SARS-CoV-2 spikes, suggesting that spike density may not factor as much in this circumstance. On the other hand, spike density could affect other important interactions, such as antibody neutralization.

Reviewer 1 Minor Comment 4: This is totally an aside/out of scope, but the last paragraph made me think about long-term technology development – another really powerful development would be if it were possible to not only create libraries with different receptor orthologs or variants, but also somehow combine this with libraries on the viral side, for library-on-library assays in the context of full cellular infection. I’m not sure if this could be managed with some sort of second landing pad site that the viral genome can integrate into, and/or

some single-cell / droplet-based microbiology to link viral and receptor identifier barcodes. Anyway, that would obviously be a very long-term endeavor, but would be a very cool direction!

> This is a great idea, and we would love to perform such types of studies in the future. A single-cell / droplet-based technology would be needed to perform such library-on-library experiments, and due to technological and cost limitations, could likely only be performed with relatively small libraries of hundreds of combinations (and thus tens of members for each library) with existing methods. As a group keen on technology development, we will definitely try to find ways to make such experiments more practical in the coming years!

Reviewer 2 Major Comment 1: After reading the title, I was expecting some interesting analysis on how certain human populations are more resistant or more susceptible to SARS-CoV-2 infections, only to find out that this manuscript, like many other studies in the literature, was mainly about how some ACE2 mutations affect the proteins's coronavirus receptor activities. Specifically, the finding correlating the allele frequencies of the ACE2 variants and their coronavirus receptor activities, which is supposed to be most interesting point of the study, were presented in a small panel as Figure 4E. So could the authors summarize these data in a more comprehensive table or figure so that people can evaluate the risk of being infected by SARS-CoV-2 for certain populations?

> We regret the confusion experienced by the reviewer, and have altered the title to “Variants of human ACE2 differentially promote SARS-CoV and SARS-CoV-2 spike mediated infection” to better clarify the focus of the study. Notably, ~ 85% of the variants we tested in the study are possible through single-nucleotide variation. Slightly more than half of these variants have actually been observed in various genome and exome sequencing studies, albeit at extremely low frequencies. Thus, while we did not exhaustively look at human variants beyond this set of two dozen known or possible human variants, this was largely because we believe there is limited tolerated sequence variation in human ACE2, and accordingly, do not believe ACE2 variants will have profound impacts on SARS-CoV-2 entry and infection in the general population. We have created a supplementary table 1, which summarizes all of the variants tested, as well as the human variants observed in the regions of ACE2 known to interact with SARS-CoV or SARS-CoV-2 spikes, as well as their associated allele frequencies observed in the GnomAD and TOPMed BRAVO databases.

Reviewer 2 Major Comment 2: Could the authors clarify how the allele frequencies of ACE2 variants were determined and how accurate these numbers are? Also, What is the rationale or criteria for choosing the 18 out of 276 known ACE2 variants for testing in the current study?

> The allele frequencies of ACE2 variants were taken from the GnomAD and TOPMed BRAVO databases. These are aggregated whole-genome or exome data from populations, where individuals known to be affected by severe pediatric disease, as well as their first-degree relatives, have been removed. Individuals with severe disease may still be included in these datasets, although likely at frequencies equivalent to or lower than that is seen in the general population. Thus, the ACE2 variants observed in this dataset are likely largely reflective of what is there in the general population. Notably, there is a bias for individuals of european ancestry, which will skew the observed allele frequencies, though this does not change the fact that all observed ACE2 missense variants are rare overall in humans.

We have created a new Supplementary Table 1 that lists all of the ACE2 variants we tested, and denotes the rationale for why they were chosen. Four variants (K31D, Y41A, K353D, and R357A) were chosen as controls, as these variants were previously shown to affect SARS-CoV spike interaction. All remaining variants that were tested are possible by single nucleotide variation, which means they are known to exist or are likely to exist as germline variants somewhere in the world if they are compatible with life. Seven known germline variants,

spanning the entirety of the ACE2 coding sequence, were chosen based on our hypothesis that ACE2 variants located outside of the binding interface but tolerated in humans likely do not affect spike binding. Of these, G751E was chosen in particular due to its potentially disruptive nature introducing a changed glutamic acid residue in place of glycine in the transmembrane domain. 16 variants were chosen based on their proximity to the SARS-CoV-2 spike interface as determined by the 6m17 cryo-EM structure. The particular variant allele was chosen by a stepwise criteria. If a particular allele was already observed in GnomAD or Bravo, we tested that allele. If no allele was already observed, we chose one of the most non-conservative substitutions possible through single nucleotide variation at that position.

Reviewer 2 Major Comment 3: The authors made a lot of negative comments on other assays that study coronavirus/receptor interactions. However, the current manuscript mainly relies on pseudovirus entry assay and flow cytometry, both of which are less accurate than some other biochemical assays (such as surface plasmon resonance). The authors need to tone down these statements.

> We very much appreciate the utility of biochemical assays that study receptor interactions, and believe our data works alongside existing data to provide a more complete picture. We believe we express these sentiments with such nuanced passages including lines 74 to 76 “Thus, monomeric binding, while simple and quantitative, likely hides the true dynamics of spike - ACE2 avidity interactions that occur during virion attachment and entry into cells.” or lines 286 to 288 “This data helps calibrate existing ACE2 - spike binding data, so that its limitations for extrapolation to infection can be better understood and predicted.”

Reviewer 2 Major Comment 4: The authors repetitively claimed that their approaches are the most physiologically relevant because of the multivalent binding interactions between multiple spike molecules and multiple ACE2 molecules, but over-expressing ACE2 in cells is quite different from what happens in vivo (ACE2 is known to be lowly expressed in the upper respiratory tracts that SARS-CoV-2 targets). The authors need to tone down these statements.

> We do not claim that our engineered cells are physiologically relevant, though we do stress that multivalent interactions should be considered when interpreting how various factors, such as ACE2 protein sequence, affect infection. However, we do agree with the reviewer that overexpression of ACE2 is quite different from what happens in vivo, and why we carefully characterized our expression system in Figures 2 and 3 of the manuscript. In fact one of the key observations of the manuscript is that gross overexpression of ACE2 masks important but nuanced effects on receptor/virus interactions. We find that when we use constructs engineered to produce lower ACE2 levels, we are able to reveal some of the same effects that are seen with biochemical assays. Notably, the Western blotting experiments we performed for the revised manuscript show that we achieve relatively low levels of steady-state ACE2 abundance with the suboptimal Kozak cells, well below the endogenous ACE2 expression in Vero E6 cells. Thus, while we are exogenously expressing transgenic ACE2, the majority of our experiments do so at a level far below traditional overexpression.

Reviewer 2 Minor Comment 1: In the introduction, the authors forgot to include furin as one of the proteases that activate SARS-CoV-2.

> We had not originally included furin, as furin cleavage happens in the producer cell, and this cleavage event is not essential for viral entry, in contrast to ACE2 binding and making the S2’ cleavage releasing the fusion peptide. Regardless, we have added the following sentence to the revised manuscript:

Introduction, [lines 52 to 56]: “Similar to MERS-CoV but dissimilar to SARS-CoV, the SARS-CoV-2 spike possesses a furin cleavage site that separates the S1 and S2 units during virus release from producer cells [4]. While this cleavage is not essential and can be performed by host proteases in the target cell, furin cleavage

increased pathogenicity in animal models [5,6]. The first essential step for productive SARS-CoV or SARS-CoV-2 infection occurs when the spike protein uses its receptor binding domain (RBD) to interact with cell surface ACE2[2,7]"

Reviewer 2 Minor Comment 2: It seems that the authors measured the ACE2 expression in cell lysates. But to quantify the cell surface expression of ACE2, cell membrane-associated ACE2 needs to be measured.

> We utilized two different methods to assess ACE2 protein abundance. First, we used a secreted SARS-CoV-2 superfolder GFP fusion protein, expressed from a plasmid created by Dr. Erik Procko's lab at the University of Illinois. This protein recognizes ACE2 on the cell surface, although it is incapable (at least under the tested conditions) of visualizing small amounts of ACE2 on the cell surface. Thus, ACE2 levels on the cell surface were measured, albeit not fully quantitatively across the full range of expression levels tested. In cases where we had to detect small amounts of ACE2 (such as in parental HEK 293T cells or with WT ACE2 or its variants in the suboptimal Kozak setting), Western blotting was a useful choice as the protein size-separation and the ability to perform long exposures made it feasible to visualize and quantitate even very low amounts of ACE2 protein. While neither assay gives a suitable picture in isolation, we believe the combination of these two approaches provided a fairly complete picture for ACE2 abundance across the manuscript.

Reviewer 2 Minor Comment 3: Some of the cited literatures are inaccurate.

> We have reviewed our cited literature but were unable to identify which ones the reviewer deemed inaccurate.

Reviewer 2 Minor Comment 4: Isn't data a plural?

> You are correct and "data" is plural and "datum" is singular, though all of the data we discuss are either collections of experiments, replicates, samples, or data points, and thus our choice of wording in our manuscript would still seem to follow convention.

Reviewer 2 Minor Comment 5: Line 148: Purified recombinant RBD should be used, not supernatants containing the RBD.

> While studies with purified RBD would be the most quantitative, even supernatants containing the RBD can be quite informative with far less effort needed in preparing the materials before performing the experiments. We believe the consistency in cell-surface staining with RBD-containing supernants shown in Figure S1D, Figure 1D, and Figure 2C convey just how reproducible it still is. Notably, Dr. Erik Procko's group used SARS-CoV-2 RBD-sfGFP -containing supernantants to perform their ACE2 deep mutational scan, and our own use of similar supernants with their construct allows the two datasets to be more easily compared.

Reviewer 2 Minor Comment 6: Cells are "transducted" by pseudovirus particles, not "challenged". There are also several other inaccurate virology terms used in the manuscript.

> While we considered using the word "transduced", we are most familiar with this terminology in describing transfer of genetic material from one microorganism to another by using a viral agent like a lentiviral vector, thus bestowing a new function. While each of the experiments using GFP reporter pseudoviruses would be transducing cells with GFP, bestowing green fluorescence, we believe this terminology may confuse the reader, as the goal is not to turn cells green but instead to use a green fluorescent reporter as a quantitative readout for the efficiency of viral entry. To circumvent this potential point of confusion, we have opted to substitute the word "challenge" with "mixed" or "exposed":

Results [Lines 432 to 433]: “The same cells exhibited no difference to infection when exposed to either SARS-CoV or SARS-CoV-2 spike coated pseudoviruses.”

Results [Lines 165 to 167]: “We thus engineered cells overexpressing ACE2 K31D or K353D and exposed them to SARS-CoV or SARS-CoV-2 pseudovirus, including a construct where the entire ACE2 ectodomain was deleted (dEcto) to serve as a negative control (Fig 2B)”

Results [Lines 350 to 352]: “We next exposed a panel of ACE2 variants to pseudoviruses coated with the SARS-CoV-2 spike variants, and assessed whether either spike variant had altered infectivity compared to the WT spike”

Results [Lines 322 to 324]: “To assess whether the ACE2 variant reliances measured with SARS-CoV-2 pseudoviruses correlated to infection with actual, replicating SARS-CoV-2, we exposed a subset of ACE2 variant cells to the WA1 SARS-CoV-2 isolate.”

Reviewer 2 Minor Comment 7: Do these ACE2 mutations affect the peptidase activities of ACE2?

> The variants that reduce ACE2 protein abundance, such as R357A and G751E, would presumably effectively reduce ACE2 peptidase activity on cells by reducing the amount of ACE2 protein on the cell surface. Since the ACE2 peptidase active site is distinct from the surface bound by SARS-CoV or SARS-CoV-2 spike RBD, we do not believe the other protein-surface variants would reduce ACE2 enzymatic activity. Notably, Dr. Farzan and colleagues in their 2005 EMBOJ article (PMID: 15791205) showed that the ACE inhibitor MLN-4760 neither interfered with SARS-CoV spike RBD binding nor infection, supporting the observation that virus entry is independent from peptidase activity.

Reviewer 2 Minor Comment 8: The discussion is lengthy. It needs to be shortened and focus on relevant topics.

> We attempted to tidy up the discussion section, though we could not identify entire sections that could be removed without losing the relevant discussions that were important for interpretation of the manuscript.

Reviewer 2 Minor Comment 9: The results section also needs to be cleaned up to be more concise.

> We attempted to tidy up the results section, but were weary of cutting too much at the risk of losing clarity.
